# Green transformational leadership and employee organizational citizenship behavior for the environment in the manufacturing industry: A social information processing perspective

**DOI:** 10.3389/fpsyg.2022.1097655

**Published:** 2023-01-18

**Authors:** Xuhong Liu, Xuan Yu

**Affiliations:** ^1^Department of Police Management, Sichuan Police College, Luzhou, China; ^2^School of Economics and Management, Southwest Petroleum University, Chengdu, China

**Keywords:** green transformational leadership, green organizational climate, organizational citizenship behavior for the environment, environmental concern, manufacturing industry

## Abstract

The employee organizational citizenship behavior for the environment (OCBE) contributes to the improvement of the organization’s environment, its study is increasing in number. However, the psychological mechanism of promoting employee OCBE is still a missing link. Drawing on the theory of social information processing, this study seeks to establish the impact of green transformational leadership on employee OCBE and the mediating role of green organizational climate in this nexus. In addition, we have integrated environmental concerns to better explain the impact of this differentiation. The results show that: green transformational leadership has a significant positive impact on employee OCBE, and green organizational climate has a mediating effect on the impact of green transformational leadership on employee OCBE. Furthermore, environmental concern not only has a positive moderating effect on the influence of green transformational leadership on green organizational climate, but also positively moderates the impact of the influence of green transformational leadership on employee OCBE. This paper reveals the internal psychological mechanism of improving employee OCBE and provides ideas for promoting the sustainable development of enterprises.

## Introduction

With rapid economic development, resource consumption and environmental problems have become increasingly severe, with environmental protection becoming one of the most urgent social priorities throughout the world (Ones and Dilchert, 2012; Imbrogiano and Nichols, 2021). Academia discussed how to improve organizational environmental performance from formal environmental management control and informal environmental management control perspectives (Pondeville et al., 2013). Meanwhile, it is undeniable that employee organizational citizenship behavior for the environment (OCBE) is one of the important paths to environmental sustainability (Khan and Khan, 2022). OCBE as a voluntary green behavior is not explicitly recognized by the official reward system but helps organizations to carry out more effective environmental management (Boiral, 2009). When employee exhibits OCBE, they participate in voluntary environmental actions beyond work requirements (Daily et al., 2009; Ojo and Fauzi, 2020). A growing number of studies also shown that environmental management practices can only be sustainable when they are supported by the participation of employees (Paillé et al., 2013; Pinzone et al., 2015). However, the discussion on the antecedents of employee OCBE is still insufficient (Andersson et al., 2013; Nurwahdah and Muafi, 2022). This article specifically attempts to address what factors affect employee environmental organizational citizenship behavior and how they work.

Leadership is one of the main factors affecting employees’ attitudes and behaviors. Accordingly, scholars have studied different leadership styles affect employee OCBE. For example, Zhang et al. (2016) found that supervisors’ ethical leadership have a positive relationship with organizational environmental citizenship behavior, this research was echoed in a follow-up research by Khan et al. (2019) and Saleem et al. (2020); Moreover, Luu (2019, 2020) indicated environmentally specific charismatic leadership and environmentally specific servant leadership demonstrated a role in shaping employee OCBE. Nurwahdah and Muafi (2022) indicated that green transformational leadership has a significant impact on employee green organizational citizenship behavior. It should be noted that some scholars emphasized that transformational leadership could promote employee OCB better than transactional leadership or other leadership styles (Podsakoff et al., 1990, 2003). Similarly, the advantages of environmental transformational leadership are reflected in the green leadership literature (Egri and Herman, 2000; Robertson and Barling, 2017). Green transformational leadership refers to the extension of transformational leadership style to the field of environmental protection, which can motivate subordinates to exceed the expected environmental goals (Chen and Chang, 2013). This leadership style is critical to employees’ pro-environment behaviors in the workplace (Robertson and Barling, 2013). When leaders adopt democratic and open communication on matters related to the environment, employees are more willing to take environmental measures (Ramus and Steger, 2000; Ramus, 2001). Therefore, the encouragement and support of environmental protection initiatives by green transformational leaders can motivate employee to actively engage in green behaviors (Chen and Chang, 2013; Robertson and Barling, 2013). However, these studies have not been able to reveal enough about the potential mechanisms that affect employee OCBE, there is a lack of in-depth discussion on the process and boundary conditions despite the fact that existing studies have provide a preliminary insight into the role of leaders in predicting OCBE.

At the same time, most studies in the green leadership literature have focused on the perspective of normative behavior theory (Norton et al., 2014; Robertson and Carleton, 2017), the theory of planned behavior (Omarova and Jo, 2022); social exchange perspective (Daily et al., 2009; Paille and Boiral, 2013) and sociological learning theories (Khan et al., 2019; Saleem et al., 2020). These views provide a valid explanation for understanding how leadership style affects employees’ positive environmental behaviors but ignore the role of social information processing theory. Social information processing theory states that individuals must weigh their own judgments when processing different information, which can be affected by surrounding events and social interaction (Schneide et al., 1998). Therefore, when employees observe that people around them are engaged in positive environmental behaviors, they may also engage in these positive green behaviors through social interaction with leaders or colleagues out of the desire to integrate, establish or strengthen social relations with people around them, which helps to form a green organizational climate (Kim et al., 2017; Khan and Khan, 2022). Green organizational climate refers to the atmosphere formed by a series of sustainable development policies implemented by the organization, that is, employees’ common views on the organization’s environmental management policies, practices, and processes (Zientara and Zamojska, 2016). Green transformational leadership helps organization members understand the concept of environmental protection through the green guidance of organizational policies and personal behaviors (Zhou et al., 2018; Khan and Khan, 2022). This kind of leadership contributes to the formation of green organizational climate and promotes the generation of employee environmental behaviors (Khan et al., 2019). Therefore, this study selects green organizational climate as the mediator variable in explaining the impact of green transformational leadership on employee OCBE.

In addition, situational factors have a significant impact on organizational and individual behaviors and may even be embedded. The rise of new environmental paradigms has stimulated scholars’ interest in understanding how people view and pay attention to environmental issues (Zhu et al., 2020). Environmental concern represents an individuals’ concern for and understanding of environmental problems, as well as their willingness to solve problems and make efforts (Daily et al., 2009). Studies have shown that managers’ environmental awareness explains enterprises’ environmental behavior from an internal micro perspective and is an important factor influencing enterprises’ green practices (Stern and Dietz, 1994; Zhang et al., 2015). Managers with higher environmental awareness and concern tend to assume higher social/environmental responsibilities, actively deal with environmental problems, and produce environmentally friendly behavior (Tseng et al., 2013). Omarova and Jo (2022) proposed environmental awareness mediated the relationship between environmental leadership and employee pro-environmental behavior from the theory of planned behavior. But Bamberg (2003) believes that environmental concern should be an indirect factor rather than a direct factor in the process of influencing environmental behavior. Because it works by influencing the production of context-specific cognition. This view is echoed in the work of Saleem et al. (2020), so as Cao and Chen (2019). Therefore, this paper selects environmental concern as a moderating variable to explore the role of green transformational leadership in the green organizational climate and employee OCBE.

**Figure 1:**
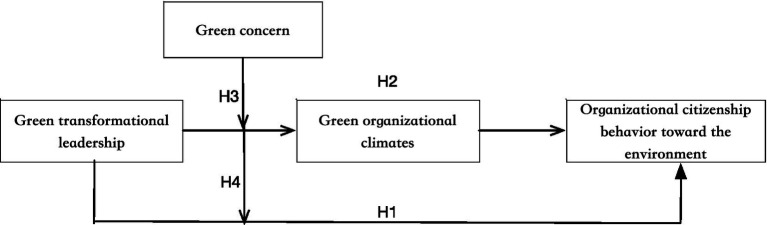
The proposed model of the study.

Based on the above discussion, this study took green organizational climate as the mediator variable, introduced the moderating variable of environmental concern, respectively, analyzed and investigated the impacts of green transformational leadership on employee OCBE, to provide better guidance for theoretical research and organizational practice. The hypothetical framework is shown in Figure 1.

## Theoretical background and hypothesis

### Green transformational leadership and employee OCBE

OCBE is a kind of spontaneous out-of-role behavior (Boiral, 2009; Paillé et al., 2013; Khan and Khan, 2022), that is usually not rewarded or required by the formal system of an organization. Thus, it is a kind of voluntary behavior outside the work tasks and responsibilities (Ehrhart and Naumann, 2004). However, it can effectively supplement the defects and deficiencies of the formal environmental management system (Boiral, 2009; Daily et al., 2009) to promote the green development of an organization. Green transformational leadership is an extension and application of transformational leadership theory in the field of environmental responsibility. It mainly focuses on encouraging and supporting proactive environmental protection measures to motivate individuals and organizations to collectively produce environmental behaviors beyond expectations and achieve environmental goals (Chen and Chang, 2013; Robertson and Barling, 2013; Saifulina and Carballopenela, 2017). According to its characteristics and connotations, Robertson and Barling (2013) divided it into four aspects: environmental idealized influence, environmental inspirational motivation, environmental intellectual stimulation and environmental individualized consideration Robertson.

Green transformational leadership influences employee voluntary pro-environment behaviors through internal motivations and emotional states (Graves et al., 2013; Robertson, 2017). Specifically, green transformational leaders can be an example in organizations for employees to get used to a working system that can care about the environment (Srour et al., 2020), exert an idealized influence of environmental protection, advocate and practice environmental protection concepts to create a “role model” effect, and shape behaviors that are consistent with environmental vision (Robertson and Barling, 2013). Importantly, in these processes, environmental inspirational motivation can convey signals to employees that environmental protection must be prioritized, as it can build confidence environmental passion (Li et al., 2020) which can inspire employees to think about environmental issues (Robertson and Barling, 2013). At the same time, green transformational leaders provide necessary resources (organizational structure, staffing and technical support, etc.) in the workplace to develop employees’ potential and skills, it related to environmental protection and improve their ability to think of solutions to environmental problems through environmental intellectual stimulation (Robertson and Barling, 2013; Zhou et al., 2018). In addition, through personal care for environmental protection, employees can be empowered to undertake challenging environmental protection work and responsibilities (Robertson and Barling, 2017), thus enhancing their control (Afsar et al., 2019), and the opportunity/ability of OCBE. Based on the abovementioned ideas, the following hypothesis is proposed:

*H1*: Green transformational leadership will be positively associated with employee OCBE.

### The mediating role green organizational climate

Green organizational climate refers to the atmosphere formed by a series of sustainable development policies implemented by the organization, that is, employees’ common views on the organization’s environmental management policies, practices, and processes (Zientara and Zamojska, 2016). Meanwhile, green organizational climate shapes an implicit code of conduct. It shows which behaviors are effective and appropriate, and which do not meet the expectations/requirements of the organization (Norton et al., 2017). This comes from the precondition of atmosphere formation. Organizational climates are formed by individuals in interactive learning, and in this process, the formation organizational climate also depends on the behaviors of employees’ superiors or managers and how they explain the framework of policy formulation to the former (Kuenzi and Schminke, 2009). Following the conceptualization of social information processing theory, individuals often receive multiple information sources in the workplace and seek to explain uncertain information around them through social interaction (Schneide et al., 1998). When employees face the dilemma of how to balance economic goals and environmental goals, green change leaders can guide employees to clearly perceive the organization’s environmental value orientation and strategic goals (Robertson and Carleton, 2017; Zhou et al., 2018). Specifically, in organizational environmental management practices, green transformational leaders convey environmental values through the issuance of policy statements, assignment of environmental tasks to subordinates, and explaining the reasons for the specific plans of the organization (Zientara and Zamojska, 2016). A positive green cultural climate can be created among different levels by building a common vision within the group (Alt and Spitzeck, 2016). Therefore, the formation of green organizational climate is influenced by the attention and support of managers to environmental management policies (Kuenzi and Schminke, 2009). At the same time, green transformational leadership has a demonstration effect on employees’ behaviors, providing organizational members with charisma and the representation of direct observational learning. This can shift their focus from the one-way influence of formal leadership, enabling them to feel the collective influence of the organization.

Green organizational climate can induce employee green behavior by allowing them to conduct social interactions (staff with colleagues, staff and leadership) with extra effort and in ways that are consistent with environmental protection behaviors (Pondeville et al., 2013; Khan et al., 2019). On the one hand, the process of social interaction will strengthen employees’ social learning, through their actions and behaviors, and participate in environmental protection behaviors, such as recycling, energy conservation and encouraging others to protect the environment (Khan et al., 2019). On the other hand, the atmosphere of implementing environmental protection practices can affect employees’ environmental resonance (Zientara and Zamojska, 2016; Biswas et al., 2022). These can promote the implementation of employee green behaviors by green transformational leadership establishing a common environmental vision among members (Graves et al., 2013; Alt and Spitzeck, 2016). This comes from the environmental values and environmental protection practices displayed by green transformational leadership can be easily perceived by employees. Further, a transmission of psychological resources after information processing can be internalized. Such an influence process can create an atmosphere that encourages employee OCBE and the formation of a relatively consistent environmental orientation in the group (Saez-Martinez et al., 2016). This also can promote mutual dependence and learning among members, thus motivating their colleagues to produce OCBE (Ramus and Steger, 2000; Zientara and Zamojska, 2016). Based on this information, the following hypothesis is proposed:

*H2*: Green organizational climate has a mediating effect on the relationship between green transformational leadership and employee OCBE.

### The moderating role of environmental concern

Environmental concern represents an individuals’ concern for and understanding of environmental problems, as well as their willingness to solve problems and make efforts (Daily et al., 2009). Under the new environment paradigm, managers’ inherent values and beliefs are different, and their choices of corporate environmental behaviors may also vary. Managers with a higher degree of environmental concern are more likely to realize the seriousness and urgency of environmental problems. They are also more inclined to emphasize common environmental goals and emotions in their work, thus improving collective environmental awareness among organization members and intensifying the formation of green organizational climates (Ardoin et al., 2015). This can also be achieved by initiating and participating in environmental cooperation as well as sharing and exchanging relevant environmental knowledge through open discussion. All these efforts can create a more pro-environment organizational climate (Norton et al., 2017). At the same time, high environmental concern means that managers are better able to actively acquire and master relevant information to help the organization better deal with uncertainties/risks (Cao and Chen, 2019). Environmental policies formulated under this condition are more likely to gain support and trust, and thus more likely to form a consensus on environmental issues (Todaro et al., 2019; Luu, 2020). On the contrary, low environmental concern makes it difficult for organization members to feel its firm determination to solve environmental problems, and they may even detect hesitation. It is not conducive to the formation of a strong organizational climates (Ojo and Fauzi, 2020). Thus, the following hypothesis is proposed:

*H3*: The higher its environmental concern, the stronger the impact of green transformational leadership on green organizational climate.

Accordingly, employees will selectively engage in behaviors supported by leaders due to the latter’ environmental concern. In turn, this will directly affect whether employees are willing to make additional environmental protection efforts (Daily et al., 2009). Research shows that attitude is a direct predictor of actual behavior, and those managers who pay more attention to the environment are more likely to change from being in the initial consciousness stage to possessing deeper values. Such leaders take the initiative in environmental protection behavior by setting an example and inducing a role model effect in their work (Boiral et al., 2015). To a certain extent, it is easier to form descriptive norms, convey strong environmental value orientation to organization members, and clarify to the employees the corresponding behavior scope, which can motivate them to take the initiative in environmental protection efforts (Dumont et al., 2017). Based on such information, the following hypothesis is proposed:

*H4*: The higher the environmental concern, the stronger the influence of green transformational leadership on OCBE.

## Materials and methods

### Participants and procedures

The data collection was conducted in two ways: on-site and online. Questionnaires were collected from manufacturing enterprises in Southwest and Eastern China engaged in electronics and information, biotechnology, environmental protection and other high-tech fields. Middle and senior managers and grass-roots managers related to environmental management were selected as the study’s subjects. Considering the availability and validity of data, the nearest sampling method and target sampling method in non-probability sampling were mainly used to distribute questionnaires. First, a small sample test was conducted, and items with factor loadings lower than 0.5 were removed before the formal questionnaires were issued. Finally, 377 questionnaires were obtained, from which 312 valid samples were retained after excluding those with missing information samples, abnormal samples, and samples of industries and positions that did not conform to the scope of the research object.

Among the respondents, male managers accounted for 73.72%, middle managers accounted for 39.42%, and top managers accounted for 26.28%. Furthermore, 76.92% had a bachelor’s degree or above. Regarding ownership of the business, state-owned enterprises accounted for 22.76%. Among the established years of enterprises, 74.68% of the enterprises have been established for 7 years or more, 13.46% for 3–7 years, and 11.86% for less than 3 years. Regarding the scale of enterprises, companies with less than 100, 101–500, 501–1,000, and over 1,000 employees comprised 28.53, 28.53, 28.85, 11.22, and 31.41% of the sample, respectively.

### Measures

In this study, a foreign maturity scale with good credibility validity in existing literature was selected as the measurement scale. After it was translated into Chinese, two English doctors were invited to back-translate the scale. After discussion with three enterprise managers, the final scale was formed. A 5-point Likert subscale was used for all scales (5 = “strongly agree” and 1 = “strongly disagree”).

### Measurement of green transformational leadership

The research scale of green transformational leadership was adopted form Robertson and Barling (2013), who used a research scale with 8 items. A sample item is “Leader communicates a clear and positive vision of the future.” Its Cronbach’s alpha value was 0.928, the composite reliability (CR) was 0.928, and the average variance extracted (AVE) was 0.618.

### Measurement of OCBE

As this paper studied the OCBE of employees at the organizational level, the research scale of OCBE was adopted from Pinzone et al. (2016) and appropriately revised. Eight items were used to measure the contents of environmental active behavior, environmental citizen participation behavior and environmental help behavior. A sample item is “Employees stay informed on environmental activities in the Trust.” Its Cronbach’s alpha value was 0.953, the CR was 0.953, and the AVE was 0.717.

### Measurement of environmental concern

The 4-items research scale of environmental concern was adopted by Kim et al. (2017). In this study, the correlation between the item “plants and animals have as many rights as human beings” and the total score was 0.355. This indicates that this item has weak homogeneity with the overall scale, so it is considered to be removed. Cronbach’s Alpha value of the scale is 0.813, and CR was 0.782, the AVE was 0.545.

### Measurement of green organizational climates

The research scale of green organizational climates was adopted by Norton et al. (2014) with four items, and the managers evaluated the overall environment of the enterprise’s green organization. A sample item is “our company is worried about its environmental impact.” Cronbach’s Alpha value of the scale was 0.895, and CR was 0.900, the AVE was 0.695.

### Control variables

It has been found that differences in enterprises’ size and nature as well as the managers’ age and educational background have varying impacts on enterprises’ environmental practices. To this end, we took it as a control variable and controlled it in the regression analysis.

### Data analysis

The main statistical software used in this study were SPSS23.0 and AMOS24.0. AMOS is mainly used for confirmatory factor analysis (CFA) and model testing. In the first stage, the polymerization validity of the study was explained by factor load, complex reliability (CR), and average variation extraction (AVE), in accordance with the study of Fornell and Larcker (1981). Factor loading and AVE must be greater than 0.7 and 0.5, respectively, to indicate that the polymerization validity is relatively ideal. Moreover, the correlation coefficient of variables must be less than the square root of AVE to indicate that the discriminant validity is ideal. We chose chi-squared fit statistic (χ2/df); absolute fitting indexes, including the root mean square error of approximation (RMSEA); the standardized root means square residual (SRMR); and the relative fitting indexes, including the comparative fit index (CFI) and the Tucker–Lewis Index (TLI), as the basis for the model fitting test. In the second stage, the relationship between variables was analyzed by hierarchical regression method, and the mediating effect was further verified referred to Hayes (2015). To enhance the robustness of the effect test, the PROCESS macro plug-in by Hayes (2015) was used in this study to test the mediation effect. If the 95% confidence interval (CI) does not include zero, the mediating effect is significant.

## Results

### Descriptive statistics

Table 1 presents the descriptive statistics and correlations for the study variables. The mean and standard deviation of each variable show at the data structure is good and that there is no violation of the normal distribution hypothesis.

**Table 1:** Descriptive statistics and correlations of study variables.

Constructs	1	2	3	4	5	6	7	8
1. Enterprise scale	-							
2. Enterprise ownership	−0.128*	-						
3. Gender	0.158**	0.128*	-					
4. Educational background	0.120*	−0.063	−0.110	-				
5. GTL	−0.080	0.101	0.091	−0.080	**0.786**			
6. EC	0.005	−0.014	0.074	0.054	0.100	**0.738**		
7. GOC	0.073	0.097	0.067	−0.033	0.547**	0.082	**0.834**	
8. OCBE	0.104	0.069	0.077	0.010	0.604**	0.012	0.656**	**0.847**
Mean	2.423	1.885	1.244	2.096	3.282	3.187	3.604	3.002
Standard deviation	1.227	0.631	0.430	0.772	0.898	0.991	0.936	1.114

Tests of hypotheses are two-tailed tests; *p < 0.05; **p < 0.01; GTL green transformational leadership, EC environmental concern, OCBE organizational citizenship behavior for the environment, GOC green organizational climate. The bold value is the arithmetic square root of the AVE value of each variable.

Green transformational leadership correlated moderately with green organizational climates (*r* = 0.604, *p* < 0.01) and green organizational climates (*r* = 0.547, *p* < 0.01), but is not correlated with environmental concern (*r* = 0.10, *p >* 0.05). The correlation between the study variables is moderate or weak. The correlation between the study variables is moderate or weak, which supports subsequent hypothesis testing. Supports subsequent hypothesis testing.

### Reliability and validity

Table 2 exhibits the discriminant validity. As can be seen, the Cronbach’s α and CR values of each variable are higher than 0.7, the factor loading of each item is higher than 0.7, and all the average variance extracted (AVE) value is higher than 0.5, indicating that the reliability and convergence validity of each scale are good. Moreover, the correlation coefficient between each variable and other variable in the Table 1 is less than square root of AVE for each construct, indicating that the whole measurement tool has good discriminant validity.

**Table 2:** The discriminant validity.

Constructs	No. of items	Cronbach’s alpha	CR	AVE	Square root of AVE
GTL	8	0.928	0.928	0.618	0.786
GOC	8	0.895	0.900	0.695	0.834
EC	4	0.813	0.782	0.545	0.738
OCBE	3	0.953	0.953	0.717	0.847

We used AMOS24.0 software to conduct CFA. The fitting index of each model is shown in Table 3. The analysis results indicate that the four-factor model is significantly better than other models. Its goodness of fit (χ2/df = 2.956, RMSEA = 0.079, TLI = 0.913, CFI = 0.925, and SRMR = 0.064), indicate that the entire measurement tool has good discrimination validity.

**Table 3:** Confirmatory factor analysis results.

Model	χ2/df	RMSEA	TLI	CFI	SRMR
1. GTL, GOC, OCBE, GPI	2.956	0.079	0.913	0.925	0.064
2. GTL+ OCBE, EC, GOC	3.763	0.094	0.877	0.891	0.109
3. GTL+ EC, GOC, OCBE	4.214	0.102	0.856	0.873	0.183
4. GTL + GOC+ OCBE, EC	4.196	0.101	0.857	0.873	0.177
5. GTL + GOC + OCBE+ EC	4.329	0.103	0.851	0.865	0.216

### Regression analysis

The hypothesis test of this study used the hierarchical regression method to introduce control variables, green transformational leadership, OCBE and green organizational climates into the equation. The test results are shown in Table 4. In the collinearity diagnosis results, the highest VIF value of each regression model is 2.629, indicating that the multicollinearity problem among variables is not serious.

**Table 4:** Results of hierarchical regression analysis.

Construct	GOC				OCBE			
	M1	M2	M3	M4	M5	M6	M7	M8
Enterprise scale	0.096**	0.093**	0.091**	0.123***	0.041	0.078	0.124***	0.121
Enterprise ownership	0.087	0.084	0.077	0.047	0.018	0.006	0.044	0.032
Gender	−0.024	−0.028	−0.036	−0.006	0.062	0.005	0.004	−0.012
Educational background	−0.002	−0.004	−0.005	0.055	0.037	0.055	0.060	0.058
GTL	0.575***	0.606***	0.601***	0.680***		0.407***	0.765***	0.755***
GOC					0.688***	0.474***		
EC		0.028	0.049				−0.057	−0.018
GTL × EC			0.117*					0.218***
*R*2	0.390	0.317	0.327	0.316	0.435	0.528	0.393	0.421
△*R*2	0.370	0.297	0.010	0.297	0.435	0.092	0.373	0.028
△*F*	185.597***	66.375***	4.474*	132.673***	47.182***	59.675***	93.618***	11.492***

Tests of hypotheses are two-tailed tests; *p < 0.05; **p < 0.01; ***p < 0.001.

From Model 4, it can be seen that after adding control and prediction variables, green transformational leadership can significantly and positively affect OCBE (*β* = 0.680, *p* < 0.001), Thus, H1 is verified.

The current study adopted the three-step method proposed by Baron and Kenny (1986) to verify the mediating effect. First, the significant positive impact of green transformational leadership on employee OCBE has been verified. In the second step, as shown by Model 1 in Table 4, it can be seen that green transformational leadership can significantly and positively affect green organizational climates (*β* = 0.575, *p* < 0.001). In the third step, as can be seen from Model 6, after joining green organizational climate, these significantly affect employee OCBE (*β* = 0.474, *p* < 0.001), Furthermore, green transformational leadership can still significantly and positively affect employee OCBE, but the significant decline (*β* = 0.407, *p* < 0.001), and the adjusted R^2^ increases by 0.212. Therefore, green organizational climate plays a partial mediating role between green transformational leadership and employee OCBE. Thus, H2 is verified.

Due to the limitations of the three-step mediating effect test method, this study further uses the bootstrapping analysis to verify the mediating effect. In the specific operation, PROCESS plug-in was used, the number of repeated sampling samples was set at 5000, and the 95% CI of deviation correction bootstrap was obtained. The total, direct, and indirect impact results under the mediating effect are shown in Table 5. As can be seen, the total impact of green transformational leadership on employee OCBE is 0.743 (LLCI = 0.633, ULCI = 0.852), The direct impact of green transformational leadership on employee OCBE is 0.429 (LLCI = 0.315, ULCI = 0.544), and the indirect impact of green transformational leadership on employee OCBE through green organizational climates is 0.313 (LLCI = 0.222, ULCI = 0.405). The CI does not include 0, further verifying the mediating role of green organizational climates.

**Table 5:** Bootstrapping test results of mediating effect.

Predictor	Effect	SE	Boot95%CI	
			LLCI	ULCI
Total effect (GTL → OCBE)	0.743	0.056	0.633	0.852
Direct effects (GTL → OCBE)	0.429	0.058	0.315	0.544
Indirect effects (GTL → GOC → OCBE)	0.313	0.047	0.222	0.405

Next, hierarchical regression was used to test H3. The product terms of independent variables and moderating variables, which were, respectively, standardized, were used to eliminate collinearity. The test results are shown in Table 4. As shown in Model 3, the product terms of green transformational leadership and environmental concern have a significant positive impact on green organizational climate (*β* = 0.117, *p* < 0.05), Furthermore, *R*^2^ increases from 0.317 to 0.327 in Model 2, indicating that environmental concern positively moderates the impact of green transformational leadership on green organizational climate. Thus, H3 is verified. From Model 8, it can be seen that the product terms of green transformational leadership and environmental concern have a significant positive impact on OCBE (*β* = 0.218, *p* < 0.001). Meanwhile, R^2^ increases from 0.393 to 0.421 in Model 7, indicating that environmental concern positively moderates the impact of green transformational leadership on organizational citizenship behavior. Thus, H4 is verified.

**Figure 2:**
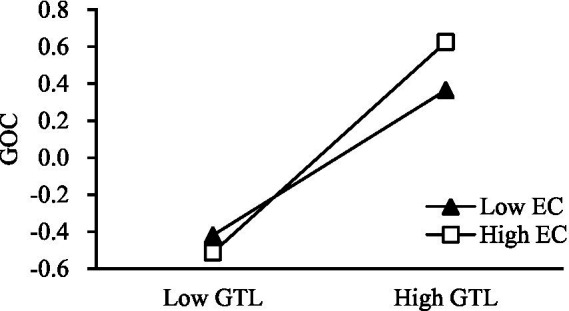
Interaction of GTL and EC on GOC.

**Figure 3:**
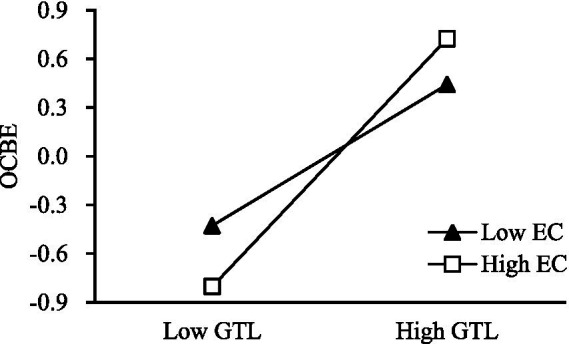
Interaction of GTL and EC on OCBE.

To understand the moderating effect of environmental concern more intuitively, this study used the simple slope analysis method, in which the average value of environmental concern is taken one standard deviation on the left and right, the sample data are divided into high and low environmental concern groups, and then the two groups are regressed, respectively. As shown in Figure 2, under the condition of high environmental concern, green transformational leadership has a stronger influence on the green organizational climate. In comparison, under the condition of low environmental concern, green transformational leadership has a weak impact on green organizational climate. This finding further verifies that environmental concern positively regulates the impact of green transformational leadership on green organizational climates. As shown in Figure 3, under the condition of high environmental concern, green transformational leadership has a stronger influence on employee OCBE. Meanwhile, under the condition of low environmental concern, green transformational leadership has a weak impact on employee OCBE. This further verifies that environmental concern positively regulates the impact of green transformational leadership on employee OCBE.

## Discussion

This study draws the following conclusions: (1) Green transformational leadership has a significant positive impact on employee OCBE. (2) Green organizational climates play a mediating role between green transformational leadership and employee OCBE, and (3) environmental concern has a positive moderating effect on the influence of green transformational leadership on employee OCBE and green organizational climates.

First, we find that green transformational leadership has a significant positive impact on employee OCBE in Chinese manufacturing enterprises. Leaders as a key factor of enterprise environmental protection, play an important role in influencing employee behavior. However, the existing research on leaders and employee OCBE cannot fully explain such a mechanism. Thus far, most studies on the relationship between green transformational leadership and employee OCBE have focused on the perspective of normative behavior theory (Norton et al., 2014; Robertson and Carleton, 2017) and sociological learning theories (Khan et al., 2019). In comparison, the main theoretical basis of this study is the theory of social information processing. According to this theory, individuals shape their attitudes and behaviors according to their social environments (Schneide et al., 1998). As an important information source of employees in the workplace, leaders are bound to have a certain impact on employee behavior. Green transformational leaders serving as “role models” can be felt by employees, which in turn, stimulates their environmental awareness and encourages them to show more proactive environmental behaviors. Therefore, based on the theory of social information processing, this study provides a new perspective for understanding how the intensity of transformational leadership motivates employee OCBE. As for management practice, on the one hand, enterprises can help existing leaders to change their minds and become green transformational leaders by training them. Through unified training and external learning opportunities, enterprises can help these managers learn from other enterprises’ experiences or relevant knowledge (Farrukh et al., 2022), understand implementation measures of green transformational leaders, and gradually change their leadership styles. Strengthen education and promotion, so that members of the organization can have great resonance with the current environmental situation (Nurwahdah and Muafi, 2022). On the other hand, enterprises can also select green transformational leaders. Enterprises can change their leadership style by looking for employees with relevant green transformational leadership characteristics from external and subordinate employees to promote their positions.

Second, green organizational climate plays a mediating role between green transformational leadership and employee OCBE. This study found that green organizational climate can explain 45% of the relationship between green transformational leadership and employee OCBE, which enables enterprises to recognize the importance of green transformational leadership on employee green behaviors. Therefore, whether employees can actively respond to environmental problems and generate OCBE not only depends on the awakening of personal environmental awareness as well as the systematic learning of environmental protection knowledge and skills, but also relies on positive green organizational climates and good demonstration effect. A good green atmosphere can help employees clearly recognize the green values and development direction of the organization, help then increase their awareness of environmental protection, and encourage them to exert extra efforts during work or non-work situations, thus enhancing the willingness of OCBE. This view is consistent with (Zientara and Zamojska, 2016). Therefore, enterprise managers must clearly indicate their attitude toward environmental issues, encourage all departments to adopt and improve their green policies and practices, and attach importance to the specific implementation of and support for green practices within the organization. For example, employees’ knowledge and awareness of the environmental protection should be strengthened through various measures, such as staff training (Khan and Khan, 2022), seminars and decoration of the actual workplace environment. These can improve employees’ active participation and encourage them to continuously study, think, and explore. Timely affirmation and praise should also be given to the positive pro-environment behaviors expected by the organization. Doing so can foster a good internal environment for stimulating employees’ proactive environmental behaviors.

Third, this study explores the important moderating effects of environmental concern on green transformational leadership of the green organization climate and employee OCBE. As mentioned above, different managers have different attitudes toward the resource support provided by an organization and its employees (Ardoin et al., 2015). However, in the existing studies, we rarely know how much leaders pay attention to environment-related issues. To some extent, this ignores the common situational perception in the interaction between leaders and employees, which is a response to the research of (Stern and Dietz, 1994). Therefore, starting from the situational factor of environmental concern, this study explores the differences in the intensity of managers’ environmental values and beliefs, as well as the differences in the formation of green organization climate and employee OCBE. The boundary conditions and control mechanism of green transformational leadership on the green organizational climate and employee OCBE are thus clarified. This work also explains the more the mechanism of the transformation of environmental concern into employee OCBE, thus extending the past research is extended (Tsai et al., 2016). In addition, organizational managers should pay more attention to external environmental pressure and learn about the successful environmental practices of competitors to maintain sensitivity to environmental problems.

Finally, our research can also be extended to government-linked companies, non-enterprise organizations, such as government organizations and non-profit organizations. In these organizations, there is also a relationship between leadership style and the active environmental behavior of organizational members. However, due to different management systems, the internal mechanisms that affect employees’ environmentally friendly behaviors within these organizations may be different. Our research provides some reference ideas.

## Conclusions and limitations

Based on of 312 managers, this paper explores the relationship between green transformational leadership and OCBE. The results show that green transformational leadership is significantly positively correlated with employee OCBE, which indicating that leaders’ transformational style can improve employee s’ environmental behavior. To further discuss the mechanism between green transformational leadership and employee OCBE, this study proposed the green organizational climates as the mediator variable of the two and combined with the internal environmental characteristics of the organization. The results reveal that almost half of the relationship between green transformational leadership and OCBE is mediated by the green organizational climates. However, the mediating effect is influenced by the intensity of leaders’ environmental concern, that is, green transformational leaders with high environmental concern are more likely to form green organizational climates inside the organization. This study concludes that employee OCBE in manufacturing enterprises is the result of a complex and comprehensive process. To encourage employees to take care of the organizational environment and save resources, the behavior style of leaders and the internal characteristics of the organization must play important roles together.

This study has some limitations that also provides a new direction for future research. First of all, the data in this study are all from individual managers’ reports. Although there is no serious homologous bias after the test, the matching sample data of leading employees can be considered in the subsequent study to minimize the impact of homologous bias and improve the accuracy of the research results. Secondly, employee OCBE is a complex process that is not only affected by leadership style and the internal environment of the organization but is also limited by external or individual resource conditions. Future studies may consider including this in the model to ensure the completeness of the research results.

## Data availability statement

The original contributions presented in the study are included in the article/supplementary material, further inquiries can be directed to the corresponding author.

## Ethics statement

Upon request, we present a letter of ethical approval from the school/institution.

## Author contributions

XL wrote the manuscript and analyzed the data. XY contributed to study design and critical revisions. All authors contributed to the article and approved the submitted version.

## Funding

This research was funded by Luzhou Key Research Base of Philosophy and Social Sciences Luzhou Social Public Security Research Center project Research on the path of urban public safety management and resilience governance (SHAQ202209), 2022 Annual project of Sichuan Party History and Party Construction Research Center: Research on the Application of “Lucky Party Building” Group Guidance in University Students’ Party Building Work (DSDJ22-14), 2022 Annual Project of Regional Public Management Informatization Research Center: Research on the path of new cloud computing service System supporting Sichuan Digital Village Construction (QGXH22-05), Chengdu social Science Planning project: The impact of industrial metaverse on the development of digital industry in Chengdu and risk prevention and control research (2022CS053).

## Conflict of interest

The authors declare that the research was conducted in the absence of any commercial or financial relationships that could be construed as a potential conflict of interest.

## Publisher’s note

All claims expressed in this article are solely those of the authors and do not necessarily represent those of their affiliated organizations, or those of the publisher, the editors and the reviewers. Any product that may be evaluated in this article, or claim that may be made by its manufacturer, is not guaranteed or endorsed by the publisher.
